# Cell Senescence as a Mediator of Age-Dependent Brain Inflammation

**DOI:** 10.1093/geroni/igab046.953

**Published:** 2021-12-17

**Authors:** Marissa Schafer, Xu Zhang, Vesselina Pearsall, Nathan LeBrasseur

**Affiliations:** 1 Physiology and Biomedical Engineering, Rochester, Minnesota, United States; 2 Mayo Clinic, Rochester, Minnesota, United States

## Abstract

Cellular senescence and inflammation are interconnected causes and consequences of tissue aging. Here, we implemented orthogonal approaches to study their interaction in steady-state mature and aged mouse brain. Using single cell sequencing, we identified a putative senescent microglial population, which increased in abundance with age and was characterized by increased expression of p16 and chemotactic senescence associated secretory phenotype (SASP) factors. Using p16-INK-ATTAC transgenic mice to eliminate p16ink4a-positive senescent cells and mass cytometry, we show that p16ink4a-positive cell targeting reduced the abundance of activated inflammatory cells in the aged female brain. Age-dependent declines in executive cognitive function were improved following transgenic p16ink4a-positive cell targeting, and executive function robustly correlated with inflammatory brain cell composition in females. Collectively, our findings demonstrate fundamental differences in the age- and sex-dependent brain inflammatory landscape and implicate p16ink4a-positive senescent cell targeting as a therapeutic strategy to attenuate age-related inflammation and cognitive decline.

